# The Role of Carbon Dots in Regulating the Periodontal Immune Microenvironment: Progress and Perspectives

**DOI:** 10.3390/ijms262110600

**Published:** 2025-10-31

**Authors:** Kun Xue, Tingting Wang, Peilei Shi, Jun Wang

**Affiliations:** State Key Laboratory of Oral Diseases, National Center for Stomatology, National Clinical Research Center for Oral Diseases, Department of Periodontics, West China Hospital of Stomatology, Sichuan University, Chengdu 610041, China; xue_kunn@163.com (K.X.); wangtt528@163.com (T.W.)

**Keywords:** periodontitis, carbon dots, immune microenvironment, antibacterial, antioxidant, immune regulation, bone homeostasis

## Abstract

As a prevalent oral chronic infectious disease, periodontitis is characterized by a complex pathogenesis, including microbial infection, host immune dysregulation, oxidative stress, and abnormal bone metabolism. Given their excellent biocompatibility, multifunctionality, and structural tunability, carbon dots (CDs) have emerged as a novel nanomaterial offering fresh approaches for the pharmacological management of periodontitis. This review systematically summarizes the application characteristics of CDs in biology and the various mechanisms in modulating the periodontal immune microenvironment. These include the roles in antimicrobial and microbiome modulation, regulation of oxidative stress balance, modulation of macrophage polarization, regulation of stem cell functions, and maintenance of bone homeostasis. The unique advantages of CDs in improving the periodontal immune microenvironment through multi-target, multi-pathway mechanisms are emphasized, thereby providing a theoretical foundation for future clinical applications.

## 1. Introduction

Periodontitis, a chronic inflammatory condition that serves as the chief culprit of tooth loss in the adult population, is also identified as the second most significant oral health burden and the sixth most common disease globally [1]. According to data provided by the World Health Organization (WHO), approximately one billion individuals worldwide are affected by severe periodontitis, resulting in an estimated annual socio-economic burden of approximately 44 billion USD [2,3]. The pathogenesis of periodontitis is complex, initiated primarily by plaque biofilms and bacterial virulence factors [4]. Sustained stimulation by plaque biofilms disrupts the host periodontal immune microenvironment, thereby inducing chronic inflammatory responses and progressive tissue destruction [4]. Traditional treatments for periodontitis primarily focus on plaque biofilm elimination through mechanical debridement of plaque and calculus, often supplemented with antibiotic therapy [5]. However, current therapeutic approaches lack the ability to directly regulate the host immune microenvironment, and conventional antibiotic treatments are prone to inducing bacterial biofilm resistance [5].

Carbon dots (CDs) have garnered considerable research interest over the past decade. As a class of zero-dimensional carbon nanomaterials, their appeal stems from a valuable set of properties, including small size, good water solubility, tunable fluorescence, ease of surface modification, and low biotoxicity [6]. The precursor flexibility for CD synthesis is remarkable, encompassing sources as varied as small molecules, polymers, food products, and pharmaceuticals [7,8,9,10]. The biological activity is primarily determined by the molecular structure of the precursor and is further modulated by surface modifications [11,12]. CDs exhibit broad potential for biomedical applications. Recent studies have reported that CDs can regulate oxidative stress levels for use in regenerative medicine and anti-inflammatory therapies [13,14,15]. CDs with near-infrared fluorescence have been utilized for targeted cancer therapy and antibacterial treatment [16,17,18,19]. Furthermore, their unique fluorescence properties have enabled applications in medical imaging [20,21,22].

As a multifunctional biomaterial, CDs have been reported in numerous studies to eliminate pathogenic microorganisms through multi-pathway and multi-target synergistic mechanisms, precisely regulate the periodontal immune microenvironment, inhibit inflammatory progression, and promote periodontal tissue regeneration. This article is dedicated to providing a systematic overview of CDs for modulating the periodontal immune microenvironment, focusing on the mechanisms and potential applications in antimicrobial activity and microbiome regulation, oxidative stress modulation, macrophage polarization, stem cell function enhancement, and bone homeostasis regulation. The insights gained from these findings are poised to inform the future development of CDs in the targeted treatment of periodontitis and periodontal tissue regeneration.

## 2. Biological Foundations of CDs in Periodontal Field

### 2.1. Application Characteristics of CDs in Biology

The unique physicochemical properties and biological activities of CDs underpin their widespread adoption in the biomedical field. CDs are defined by a suite of advantageous properties, including but not limited to superior optical characteristics, favorable physicochemical features such as small size and high water solubility, chemical inertness, straightforward surface modification, and strong resistance to photobleaching. In addition, CDs possess good biocompatibility and are not prone to accumulation in vivo [6].

#### 2.1.1. Size and Morphology

CDs are characterized by an ultrasmall, spherical or quasi-spherical structure, with sizes typically in the order of 1–10 nm. The nanoscale size facilitates the ability to penetrate biological barriers, enter cells, and interact with biomolecules. An et al. [23] highlighted that the nanoscale size of graphene oxide quantum dots (GOQDs) is a key contributor to the fundamental biological processes of cellular differentiation and tissue regeneration.

#### 2.1.2. Surface-Enriched Functional Groups

The surface of CDs is typically enriched with hydrophilic functional groups such as hydroxyl (–OH), carboxyl (–COOH), and amino (–NH_2_) groups [24]. These groups confer excellent water solubility and biocompatibility. Moreover, they provide abundant reactive sites for further surface modification and functionalization. Drug molecules, ligands, antibodies, and other agents can be conjugated to the surface of CDs through covalent bonding or non-covalent interactions, enabling targeted delivery or the integration of multiple functionalities [25].

#### 2.1.3. Unique Optical Properties

One of the most distinctive features of CDs is the unique photoluminescence (PL) mechanism [6,25]. CDs possess extensive ultraviolet–visible (UV–Vis) absorption characteristics, leading to subsequent fluorescence in the visible light range. The emission mechanism is complex and may involve a synergistic effect of various factors, such as quantum confinement effects, surface states, molecular states, and crosslinking-enhanced emission [26]. The photoluminescence of CDs, including its emission wavelength and intensity, can be tailored through a variety of strategies encompassing precursor design, synthesis condition adjustment, surface functionalization, and heteroatom incorporation [27]. This enables emissions ranging from blue to red. Compared to conventional organic dyes, CDs generally offer superior optical stability and resistance to photobleaching. These properties collectively position CDs as ideal materials for bioimaging and fluorescence-based sensing applications [28].

#### 2.1.4. Enzyme-Mimicking Activity

Many CDs, particularly those doped with specific elements, have been shown to exhibit enzyme-like activities. They can function as potent antioxidants, mimicking enzymes like superoxide dismutase (SOD), catalase (CAT), and glutathione peroxidase (GPx), while also demonstrating oxidoreductase-like activities including oxidase (OXD) and peroxidase (POD) [29,30]. This capacity to selectively generate or scavenge reactive oxygen species (ROS) positions CDs as a versatile agent for managing oxidative stress-related pathologies and combating bacterial infections.

#### 2.1.5. Excellent Biocompatibility

Within a defined concentration range, CDs have found low cytotoxicity toward various cell types, including fibroblasts, endothelial cells, and stem cells [10,31,32,33]. Their nanoscale size, combined with surface hydrophilic functional groups, facilitates good dispersion and physiological stability, reducing aggregation and minimizing non-specific adsorption and immunogenic responses [34,35]. Additionally, through surface modification such as PEGylation, CDs can be endowed with improved attributes, most notably lower immunogenicity, longer in vivo circulation half-life, and decreased non-specific clearance [24,36].

#### 2.1.6. Favorable Metabolic Properties

Nanomaterials introduced into the body—via inhalation, ingestion, dermal absorption, or intravenous injection—may pose potential risks to systems such as the cardiovascular and respiratory systems [37]. However, current evidence suggests that many small-sized CDs can be rapidly excreted through renal pathways, thereby shortening their retention time and reducing potential toxicity [6]. Nevertheless, the in vivo metabolic fate of CDs is not uniform but is a function of their specific physicochemical characteristics, including preparation method, surface properties, and size.

### 2.2. Classification and Preparation of CDs

Rather than a single substance, CDs represent a diverse collection of carbon nanomaterials, whose classification is principally based on structural features and precursor sources. The primary categories include carbon quantum dots (CQDs), graphene quantum dots (GQDs), carbon nanodots (CNDs), and carbonized polymer dots (CPDs) [6,38,39,40]. These various types of CDs exhibit differences in physicochemical properties and biological activities. Such diversity enables the tailored design and synthesis of CDs to meet specific biomedical and therapeutic requirements.

The synthesis of CDs primarily falls into two distinct categories: the “top-down” and “bottom-up” approaches [41]. In the top-down approach, nanoscale CDs are derived from the physical or chemical fragmentation of bulk carbon materials, including graphite, carbon nanotubes and carbon fibers [42]. However, this method often results in low yields and broad particle size distributions [42]. Common techniques include laser ablation [43,44,45,46,47], arc discharge [48,49,50], electrochemical oxidation [51,52,53,54], and sonochemical treatment [55,56,57]. In contrast, small organic molecules are utilized as the primary precursors in the bottom-up approach, where they undergo polymerization, carbonization, or related reactions to form CDs [40]. This strategy offers several advantages, including the use of inexpensive and readily available raw materials, mild synthesis conditions, scalability, and tunable surface functionalities [40,41]. It is currently regarded as the most promising and widely adopted method for CD synthesis. Common bottom-up methods include hydrothermal synthesis [58,59], microwave-assisted synthesis [60,61,62], pyrolysis [63,64,65], and template-assisted techniques [66,67].

Different synthesis routes impart distinct structures, surface functional groups, and optical properties to CDs, which in turn affect its behavior and biological activity. CDs synthesized from biomass frequently display favorable cytocompatibility alongside intrinsic antioxidant and modest antibacterial activity, while also benefiting from low cost and renewable feedstocks [68,69,70]. Heteroatom doping (N, S, P) or controlled incorporation of low loadings of transition metals (e.g., Cu, Fe, Bi) during synthesis has been shown to endow CDs with enzyme-mimetic activities (SOD-, CAT-, POD-like) or Fenton/near-Fenton catalytic behavior, thereby enabling potent ROS regulation and ROS-mediated antibacterial effects [71,72]. Post-synthetic surface engineering remains a versatile tool. Surface PEGylation is routinely used to reduce nonspecific protein adsorption and improve in vivo tolerance, whereas covalent grafting of cationic groups (including quaternary ammonium moieties) or conjugation of antimicrobial peptides significantly enhances electrostatic and hydrophobic interactions with bacterial membranes to promote membrane disruption [73,74]. When pharmacologically active precursor drugs are used, the resulting CDs retain the therapeutic functions of the precursor compound while introducing new optical, catalytic, or targeting properties, enabling multifunctional synergy and performance enhancement [75].

Recently, continuous hydrothermal flow synthesis (CHFS) has emerged as a promising scalable approach for producing CDs with uniform size distribution, high yield, and reproducible physicochemical properties [76]. This method allows precise control of reaction parameters such as temperature, residence time, and precursor composition, thereby facilitating the simultaneous incorporation of dopants or bioactive molecules during synthesis [77]. CHFS not only enables consistent batch-to-batch quality but also supports the generation of multifunctional CDs with combined antibacterial, antioxidant, and osteoinductive activities. Such a controllable synthetic strategy is critical for the precise modulation of the periodontal immune microenvironment. Finally, formulation into local delivery vehicles (e.g., injectable hydrogels, microspheres) has been shown to concentrate CDs at periodontal sites, prolong residence time, and reduce systemic exposure [78,79].

## 3. Mechanisms of CDs in Regulating the Periodontal Immune Microenvironment

### 3.1. Antimicrobial and Microbiome Modulation

**Figure 1:**
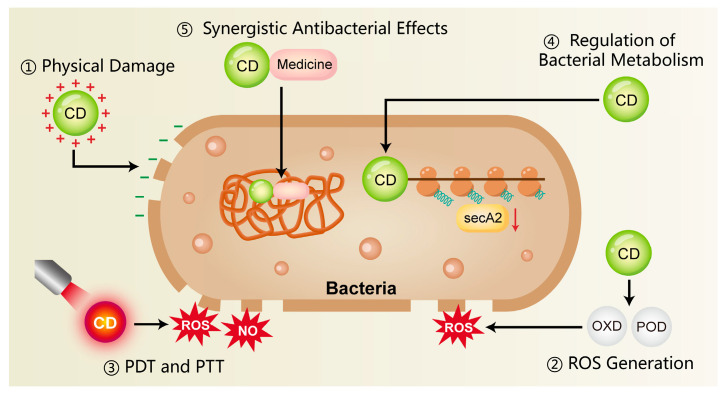
The antimicrobial mechanisms of CDs include surface-mediated electrostatic interactions, the ability to produce ROS, and their application in photothermal and photodynamic therapies. In addition, CDs can influence bacterial metabolism and act synergistically with conventional antibiotics, thereby reducing the development of antimicrobial resistance. CD, carbon dots; ROS, reactive oxygen species; NO, nitric oxide; PDT, photodynamic therapy; PTT, photothermal therapy; OXD, oxidase; POD, peroxidase.

The onset and progression of periodontitis are fundamentally linked to a state of dysbiosis in the periodontal pocket, which triggers a dysregulated host inflammatory response. *Porphyromonas gingivalis (P. gingivalis)*, recognized as a major keystone pathogen, plays a pivotal role in disrupting the periodontal immune microenvironment and impairing tissue homeostasis [80,81,82]. This pathogen evades host immune responses and triggers inflammation through various virulence factors, including proteases, lipopolysaccharides (LPS), and outer membrane vesicles (OMVs) [80,81]. CDs represent a novel strategy for periodontitis management by targeting pathogenic bacteria directly and modulating the oral microbiome towards a healthier state, such as Figure 1 and Table 1.

#### 3.1.1. Physical Damage

Bacterial cell membranes are typically negatively charged, whereas CDs often possess surface amino or other cationic functional groups that promote rapid electrostatic binding to bacteria. This lead to a loss of surface charge neutrality and subsequent destabilization of the cell membrane [90,91]. In addition, the nanoscale size of CDs facilitates close physical interactions with bacterial membranes. This disruption of membrane integrity triggers the efflux of intracellular components, thereby causing bacterial death [92]. Liu et al. [83] synthesized copper-doped carbon dots (Cu-CDs) that exhibited a pronounced interaction with LPS and peptidoglycan (PGN), displaying broad-spectrum antimicrobial action against prevalent Gram-positive and Gram-negative bacteria in the oral cavity. Research on the antimicrobial capabilities of CDs has advanced into areas far beyond oral infections, with their efficacy being actively investigated in diverse pathological contexts. For instance, Jian et al. [84] developed spermidine-derived carbon quantum dots (CQDSpds) for the treatment of bacterial keratitis. These CQDs, characterized by a small particle size (~6 nm) and high positive surface charge (ζ-potential ≈ +45 mV), enhanced the interaction with bacterial membranes and caused severe membrane disruption. Notably, this bactericidal mechanism showed broad-spectrum efficacy without apparent species selectivity, offering valuable insights for antimicrobial strategies in periodontitis.

#### 3.1.2. ROS Generation

Certain CDs mediate the production of ROS, notably superoxide anions and hydroxyl radicals, upon light exposure or under specific microenvironmental conditions. These ROS, whether produced endogenously or exogenously, can induce oxidative damage to bacterial proteins, lipids, and DNA, thereby inhibiting bacterial proliferation or directly leading to bacterial death [25,93,94]. Xie et al. [85] developed melatonin-derived carbon dots (MCDs) that significantly induced intracellular ROS production in bacteria within 30 min of co-incubation. Subsequent analysis revealed a marked increase in bacterial membrane permeability, indicating severe membrane disruption and confirming potent antibacterial activity. Similarly, Cu-CDs synthesized by Liu et al. [83] exhibited OXD-like and POD-like activities at 37 °C, catalyzing ROS production for direct bactericidal action. Studies have found that Cu-CDs possess a higher affinity for H_2_O_2_ compared to undoped CDs, and copper doping markedly enhances their enzyme-mimicking activity and catalytic efficiency.

#### 3.1.3. Photothermal Therapy and Photodynamic Therapy

A fundamental principle of photodynamic therapy (PDT) is that a photosensitizer (PS), upon irradiation with specific wavelength light, produces ROS—e.g., singlet oxygen, superoxide anions (·O_2_^−^), and hydroxyl radicals (·OH)—to achieve a therapeutic effect [95,96]. These ROS induce oxidative damage in microbial cells, primarily targeting and disrupting essential structures such as membranes, proteins, and nucleic acids. However, the short half-life of ROS often impedes the full therapeutic potential of PDT, thus requiring its combination with other antimicrobial strategies [96]. Pourhajibagher [86] synthesized a graphene derivative-based curcumin nanocomposite (GQD-Cur) for the treatment of periodontitis. Upon activation by PDT, GQD-Cur significantly enhanced antibacterial activity against *P. gingivalis, Aggregatibacter actinomycetemcomitans (A. actinomycetemcomitans)* and *Prevotella intermedia (P. intermedia)*.

In addition, some CDs possess strong near-infrared (NIR) absorption properties and are capable of converting light energy into thermal energy upon NIR irradiation. This photothermal effect induces localized hyperthermia, resulting in protein denaturation and bacterial cell death, which called photothermal therapy (PTT) [93,94,97]. Chu et al. [87] developed a NIR-responsive carbon dot platform (Cu-RCDs-C35) that achieved triple antibacterial synergy—photothermal, photodynamic, and quaternary ammonium-based effects—initiated by a single NIR light source. The elevated local temperature disrupted bacterial membrane integrity and effectively eradicated periodontal pathogens.

#### 3.1.4. Regulation of Bacterial Metabolism

CDs may exert antibacterial effects by binding to intracellular bacterial enzymes, particularly those involved in respiratory chains or metabolic pathways. This interaction can inhibit enzyme activity, disrupt physiological metabolism, and ultimately lead to bacterial death [72,98]. Xie et al. [85] reported that melatonin-derived carbon dots (MCDs) exhibited antibacterial activity by suppressing the expression of secA2, a key protein in the bacterial secretion system. Inhibition of secA2 impaired the secretion of virulence factors and reduced bacterial survival. Similarly, Li et al. [82] developed bismuth-doped carbon dots functionalized with structure-modified berberine (BiCD-Ber). These nanomaterials neutralized the gingipain virulence factors of *P. gingivalis* through the action of bismuth ions, significantly attenuating its pathogenicity. Furthermore, BiCD-Ber was incorporated into a hydrogel system responsive to pathogenic bacterial metabolites, enabling targeted drug release. This platform effectively eliminated periodontitis-associated pathogens and restored microbial homeostasis within the periodontal environment.

#### 3.1.5. Synergistic Antibacterial Effects

CDs have been shown to exert synergistic effects when combined with traditional antibiotics or antimicrobial peptides, thereby reducing the required dosage and limiting the development of drug resistance. For example, carbon dots derived from *Lactobacillus acidophilus* (L-C-dots) demonstrated dual capabilities, not only conferring marked antibacterial efficacy against carbapenem-resistant *Klebsiella pneumoniae* isolates, but also coupling with the effective suppression of biofilm formation [88]. Moreover, a synergistic and additive profile was observed for L-C-dots when used alongside the antibiotic meropenem, resulting in a reduced minimum inhibitory concentration (MIC) for both agents.

In addition to direct synergy, CDs can function as carriers for antimicrobial drugs, enabling targeted delivery to sites of infection and enhancing local drug accumulation [99]. For instance, CDs extracted from wheat bran were used as a novel drug delivery system to transport antibiotics more effectively to infected tissues, thereby improving antibacterial efficacy [89]. The multifunctionality of CDs positions them as a compelling strategy for the combined therapy of periodontal infections.

### 3.2. Inhibition of Oxidative Stress

**Figure 2:**
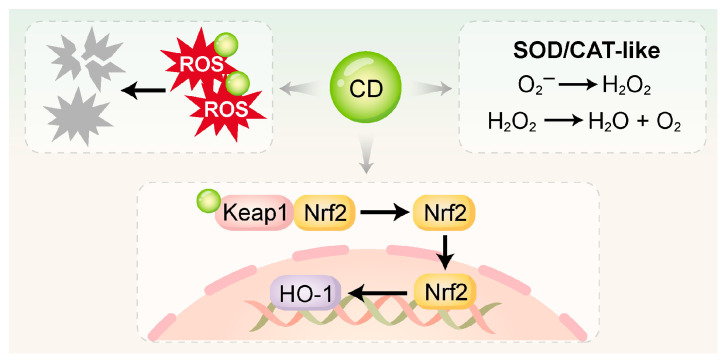
CDs directly scavenge ROS via surface functional groups and nanostructures, and mimic the activities of SOD and CAT to catalyze ROS into H_2_O_2_, H_2_O, and O_2_. Additionally, CDs can activate antioxidant pathways such as Keap1/Nrf2 and Nrf2/HO-1 to enhance intracellular redox defense. SOD, superoxide dismutase; CAT, catalase.

Oxidative stress plays a pivotal role in the pathogenesis of periodontitis. During immune responses, neutrophils and macrophages produce abundant ROS as a primary defense mechanism against pathogens [100]. However, when the homeostasis between ROS production and endogenous antioxidant defenses is compromised, a surplus of ROS accumulates, causing oxidative injury to host cells. This damage includes lipid peroxidation, protein oxidation, and DNA fragmentation, thereby amplifying inflammation, impairing mitochondrial function, and promoting cellular senescence and apoptosis [100,101]. The oxidative microenvironment not only directly injures periodontal tissues but also triggers the secretion of pro-inflammatory cytokines [102,103]. This self-perpetuating cycle drives the progressive breakdown of alveolar bone and loss of connective tissue [104]. CDs exert antioxidant effects through multiple mechanisms such as Figure 2 and Table 2.

#### 3.2.1. Direct Free Radical Scavenging

The surfaces of CDs are characterized by an abundance of functional groups, notably –COOH, –OH, and –NH_2_ [107]. Their internal conjugated structures enable them to function as electron donors or acceptors. These properties allow CDs to directly neutralize highly ROS, including superoxide anions and hydroxyl radicals, by converting them into non-toxic products [108,109]. Xin et al. [59] synthesized melatonin-derived carbon dots (MT-CDs) via a hydrothermal method, which exhibited strong ROS-scavenging activity. Without requiring additional surface modification, MT-CDs demonstrated a pronounced capacity to modulate intracellular ROS concentrations. This effect was attributed to the intrinsic antioxidant properties of melatonin combined with the unique structural characteristics of the CDs.

#### 3.2.2. Enzyme-Mimetic Activity

Many CDs—particularly those doped with specific metal ions or heteroatoms—exhibit enzyme-mimetic activities, enabling them to serve as functional analogues of endogenous antioxidant enzymes like SOD and CAT.

SOD-like activity: CDs can catalyze the dismutation of superoxide anions into hydrogen peroxide [30]. Wan et al. [105] synthesized dexamethasone-derived carbon dots (DCDs) via a hydrothermal method, integrating the pharmacological activity of dexamethasone with the antioxidant functionality of CDs. The DCDs not only scavenged hydroxyl radicals (·OH) directly but also exhibited SOD-like activity. Under inflammatory conditions, they further drove osteogenesis in rat bone marrow mesenchymal stem cells (rBMSCs).

CAT-like activity: CDs serve as a catalyst to mediate the decomposition of hydrogen peroxide into water and oxygen. Cu-CDs synthesized by Liu et al. [83] had CAT-like activity, efficiently converting H_2_O_2_ (4 mM) into H_2_O and O_2_ under physiological conditions while maintaining stability across a wide pH range. Similarly, Shi et al. [101] developed mitochondria-targeted CDs supported by Prussian Blue (CD-PB-TPP), which effectively eliminated excessive intracellular ROS. Through its dual SOD/CAT-mimetic activities, CD-PB-TPP effectively restored the mitochondrial and redox balance in nucleus pulposus cells.

#### 3.2.3. Activation of Endogenous Antioxidant Pathways

In addition to direct ROS scavenging, certain CDs have been shown to activate endogenous antioxidant defense systems, particularly the Nrf2/HO-1 signaling pathway. Nrf2 functions as a master regulatory factor in the cellular antioxidant response. Upon oxidative stress, it activates and translocates into the nucleus, where it orchestrates the transcription of genes encoding antioxidant and detoxifying enzymes [110,111]. This enhances the cell’s intrinsic ability to neutralize oxidative damage. Xin et al. [59] reported that MT-CDs exerted antioxidant effects by activating the Nrf2/HO-1 pathway. These MT-CDs also suppressed the elevated levels of pro-apoptotic proteins Bax and caspase-3 induced by H_2_O_2_, thereby reducing apoptosis. Similarly, Liu et al. [106] synthesized a red fluorescent carbonized polymer dot derived from N-acetyl-L-cysteine (NAC-CPD), which functioned as an extracellular antioxidant with efficient ROS-scavenging ability. The NAC-CPDs modulated redox homeostasis in the periodontal microenvironment by activating the Keap1/Nrf2 pathway. Compared with direct ROS elimination, this endogenous activation strategy offers more sustained and comprehensive antioxidant protection.

### 3.3. Regulation of Stem Cell Functions

**Figure 3:**
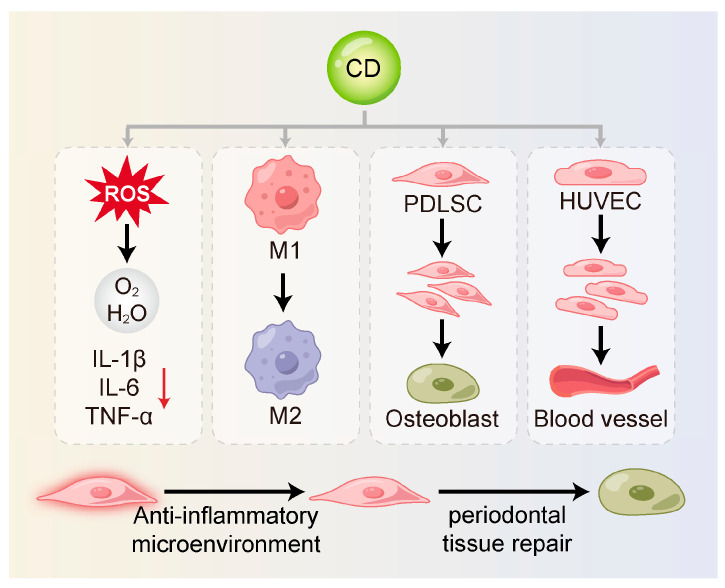
CDs can restore stem cell function and support periodontal regeneration. They reduce inflammation-induced damage by scavenging ROS and suppressing pro-inflammatory cytokines. CDs also shift macrophages from the M1 to the M2 phenotype, creating an anti-inflammatory environment. In addition, they promote stem cell proliferation and differentiation and enhance angiogenesis to support tissue repair and regeneration. M1, M1-type macrophage; M2, M2-type macrophage; PDLSC, periodontal ligament stem cell; HUVEC, human umbilical vein endothelial cell.

Achieving complete regeneration of the periodontal apparatus constitutes the central objective in periodontitis management. Mesenchymal stem cells (MSCs), including periodontal ligament stem cells (PDLSCs) and dental follicle stem cells (DFSCs), gum-derived stem cells (GMSCs) and periosteum-derived stem cells are central to the processes of tissue repair and regeneration [112,113,114,115,116,117]. However, the inflammatory microenvironment associated with periodontitis—characterized by excessive oxidative stress and elevated pro-inflammatory cytokines—impairs the proliferation, migration, differentiation, and survival of stem cells, thereby hindering effective periodontal regeneration [118,119,120,121]. Therefore, modulating the immune microenvironment to preserve and boost stem cell functionality constitutes a pivotal approach for achieving periodontal regeneration [122]. At present, most of the research on CDs and periodontal stem cells is limited to bone marrow stromal cells (BMSCs) and PDLSCs, such as Figure 3 and Table 3. Furthermore, there are currently reports of CDs giving rise to a stronger osteogenic/odontogenic differentiation capacity of DPSCs, which can facilitate the regeneration of dentin–pulp complex, and this will not be elaborated further [33,123].

#### 3.3.1. Alleviation of Inflammatory Damage

By scavenging ROS and suppressing pro-inflammatory cytokines, CDs alleviate the detrimental impact of a hostile inflammatory milieu on stem cell viability and function. Xin et al. [59] found that MT-CDs promoted tissue regeneration by eliminating ROS, preserving mitochondrial integrity, and blocking the secretion of inflammatory mediators. These effects created a more favorable environment for stem cell survival and functionality. Similarly, Jiang et al. [124] synthesized carbon dots from crude extracts of purple sweet potato (CPP-CDs). These CDs significantly reduced the release of *IL-1β*, *IL-6* and *TNF-α* in cell culture supernatants. CPP-CDs also reduced LPS-induced inhibition of intracellular ATP production and ROS accumulation. This dual action protected cells from inflammatory injury and contributed to a microenvironment supportive of stem cell activity. Further mechanistic evidence indicated that CPP-CDs exerted anti-inflammatory effects through the suppression of the TLR4/NF-κB pathway activation as well as the NLRP3 inflammasome in macrophages. These pathways are known to be critical mediators of periodontal inflammation [133].

#### 3.3.2. Regulation of Macrophage Polarization

Macrophages are essential innate immune cells within the periodontal immune microenvironment [134]. They exhibit high heterogeneity and plasticity and play dual roles in both inflammation and tissue repair [135,136]. An imbalance between pro-inflammatory M1 and reparative M2 macrophage polarization is a central driver in periodontitis [137,138,139].

CDs can modulate the phenotypic shift in macrophages through direct or indirect regulation of cytokine secretion. They can inhibit M1 macrophage polarization by curbing the production of key pro-inflammatory mediators, notably *TNF-α*, *IL-6* and *IL-1β*, while concurrently reducing pyroptosis [124,140]. Zhang et al. [125] synthesized carbon dots derived from Phellodendri Chinensis Cortex (PCC-CDs), which significantly upregulated *IL-10* and *Arg-1* expression in RAW264.7 cells, thereby promoting M2 polarization. These effects may involve pathways such as Nrf2, NF-κB, PI3K/Akt and MAPK [126,127]. Moreover, Jiang et al. [128] demonstrated that CDs can directly bind to the catalytic subunit of PI3K (PIK3CD), thereby inhibiting the PI3K/Akt/mTOR pathway to enhance bacterial phagocytosis via M1 polarization.

Additionally, elevated ROS levels in inflamed periodontal tissues serve as key signals for M1 polarization [141]. Wan et al. [105] developed DCDs, which effectively scavenged ROS and facilitated the M1-to-M2 transition, highlighting their anti-inflammatory potential. Beyond inflammation resolution, M2 macrophages release anti-inflammatory cytokines (e.g., IL-10) and growth factors such as TGF-β, VEGF, and PDGF, which collectively enhance stem cell migration, proliferation, differentiation, and angiogenesis—processes essential for periodontal regeneration. These factors support the directional movement, multiplication, fate determination of stem cells, and the angiogenesis of vascular endothelial cells [142,143]. Wu et al. [34] developed a carbon-dot-crosslinked egg white hydrogel (CEWH) that successfully established an M2-macrophage-dominant immune microenvironment, thereby promoting hair follicle regeneration. This study found the potential of CDs to modulate the immune microenvironment in support of stem cell function and tissue regeneration.

#### 3.3.3. Promotion of Stem Cell Proliferation and Differentiation

CDs have been shown to directly or indirectly enhance stem cell proliferation, promote osteogenic differentiation, and support the formation of a stable and functional extracellular matrix. These properties are essential for effective periodontal tissue regeneration. Wei et al. [127] synthesized metformin-derived carbon dots (MCDs), which activated the PI3K/Akt signaling pathway under LPS-induced inflammation thus counteracting the inflammatory response and restoring the osteogenic potential of PDLSCs. DCDs developed by Wan et al. [105] not only improved the osteoimmune microenvironment through anti-inflammatory and antioxidant effects but also directly promoted the osteogenic differentiation of rBMSCs via the pharmacological action of dexamethasone. In vitro studies further confirmed that DCDs significantly upregulated osteogenesis-related genes and enhanced mineralized nodule formation under inflammatory conditions. Shao et al. [129] synthesized citric-acid-based carbon dots (CDs), which promoted the osteogenic differentiation of rBMSCs through MAPK signaling pathway, while also providing long-term cell tracking capability. Han et al. [130] reported the hydrothermal synthesis of adenosine–aspirin carbon dots (AACDs), which demonstrated intrinsic osteoinductivity by driving the osteogenic commitment of hBMSCs without the need for external inductive factors. These AACDs outperformed treatments using either adenosine or aspirin alone. Furthermore, An et al. [23] showed that graphene oxide quantum dots (GOQDs) enhanced osteogenic differentiation of hPDLSCs by modulating mitochondrial dynamics, ultimately contributing to the repair of periodontal bone defects.

#### 3.3.4. Promotion of Angiogenesis

The formation of new blood vessels constitutes a fundamental requirement for periodontal regeneration, by delivering vital oxygen and nutrients to support newly formed tissues [144]. CDs may promote vascularization either by directly stimulating the proliferation and migration of endothelial cells or by prompting M2 macrophages to upregulate the production of factors that promote angiogenesis, thereby creating a microenvironment that supports stem cell survival and differentiation. Zhang et al. [15] synthesized CDs via a one-step hydrothermal method that exhibited SOD- and CAT-like activities, effectively scavenging O_2_•– and H_2_O_2_. These CDs were shown to restore endothelial function and promote angiogenesis in human umbilical vein endothelial cells (HUVECs) in vitro. Additionally, Xu et al. [131] used polyethyleneimine (PEI)-modified graphene quantum dots (GQDs) as gene delivery vectors to transport the *pZNF580* gene into HUVECs, thereby enhancing their proliferative capacity.

### 3.4. Regulation of Bone Homeostasis

**Figure 4:**
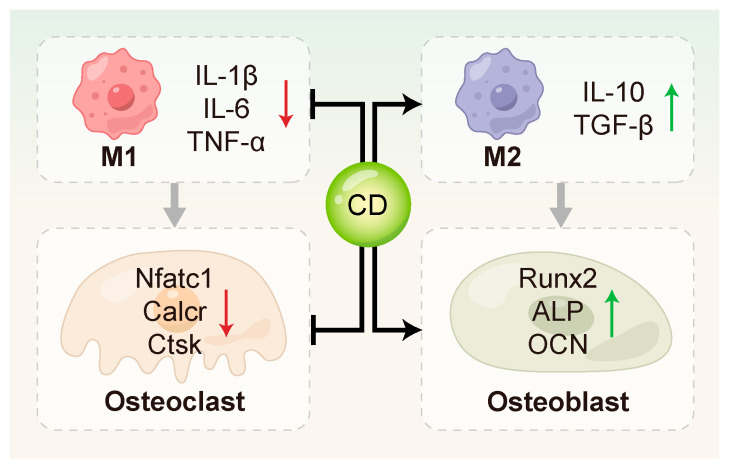
CDs regulated bone remodeling through multiple mechanisms. They can inhibit osteoclast activation by down-regulating key osteoclast-related genes such as Nfatc1, Calcr, and Ctsk, and promote osteogenic differentiation by up-regulating osteogenesis-related genes including Runx2, ALP and OCN. In addition, CDs contribute to the modulation of the bone immune microenvironment.

Periodontitis inflicts its most significant damage through the degradation of alveolar bone, an irreversible process that directly results in tooth loss [145,146]. This significantly compromises both oral function and overall quality of life [147]. A dynamic equilibrium between osteogenic and osteoclastic activity is central to the maintenance of alveolar bone homeostasis [148]. The periodontal inflammatory microenvironment promotes osteoclast activity while simultaneously impairing osteoblast function. This disruption accelerates bone resorption and undermines bone stability [149,150]. Osteoimmunology posits that the immune and skeletal systems engage in extensive crosstalk through shared signaling molecules and cellular mediators. Immune cells are now recognized as pivotal contributors to the pathological processes underlying various bone disorders, with periodontitis being a prime example [151,152]. Due to their multifaceted biological activities, CDs show great potential in modulating bone homeostasis such as Figure 4 and Table 4.

#### 3.4.1. Inhibition of Osteoclastogenesis and Activation

CDs have been shown to directly suppress the expression of key genes involved in osteoclastogenesis. Zhang et al. [78] synthesized curcumin–alendronate carbon dots (Cur-Alen CDs), which inherited the bone-homing ability of the alendronate precursor and exhibited significant inhibitory effects on osteoclastogenesis. These CDs significantly down-regulated the expression of osteoclast-related genes, including *Nfatc1*, *Calcr* and *Ctsk*. Periodontal tissues from an in vivo rat model of periodontitis exhibited a significant drop in osteoclast numbers upon TRAP staining evaluation, further confirming the anti-osteoclastic effect of Cur-Alen CDs. Additionally, Li et al. [154] first reported that photoluminescent carbon dots (PCDs) derived from malic acid could inhibit UHMWPE-induced osteoclast differentiation in vitro, thereby suppressing osteolysis.

#### 3.4.2. Promotion of Osteoblast Differentiation and Function

CDs can directly act on MSCs or PDLSCs to stimulate the expression of osteoblast-related genes, such as *RUNX2*, *OCN*, and *ALP* [10,105,127,155]. This promotes bone matrix formation and mineralization. Ren et al. [10] synthesized metformin-derived carbon dots (MCDs) that drove osteogenic progression in rBMSCs more potently than metformin alone, stimulating alkaline phosphatase activity, fostering calcium nodule deposition, and activating the expression of osteogenic genes and proteins. In addition, MCDs promoted osteogenesis through activating ERK/AMPK signaling pathway and effectively regenerating alveolar bone in a rat periodontitis model. Gao et al. [132] investigated the osteoinductive effects of graphene quantum dots (GQDs) on PDLSCs under inflammatory conditions. Their results showed that GQDs significantly up-regulated osteogenesis-related genes and enhanced mineralized nodule formation by activating the Wnt/β-catenin signaling pathway. Notably, GQDs maintained their osteoinductive potential even in the presence of LPS-induced inflammation, highlighting their potential in bone immune modulation during periodontitis. Wei et al. [155] further advanced the application of CDs in bone regeneration by developing a Nar-CuCDs/Gel composite hydrogel system. This system promoted osteogenic differentiation of rBMSCs by scavenging ROS and inducing M2 macrophage polarization, thereby optimizing the bone immune microenvironment.

#### 3.4.3. Regulation of Osteoimmunity

Osteoimmunology centers on the multicellular dialogue between immune components, notably macrophages and T cells, and bone cells such as osteoblasts and osteoclasts [156,157]. CDs can modulate macrophage function and cytokine secretion profiles, thereby indirectly influencing bone metabolism. Cur-Alen CDs synthesized by Zhang et al. [78] significantly reduced inflammatory cytokine levels and oxidative stress in RAW264.7 cells. In an in vivo rat model of periodontitis, these CDs favorably shifted bone metabolism toward regeneration, mitigating disease-induced alveolar bone loss.

Moreover, specific T cell subsets play essential roles in bone immunity. Th17 cells are known to promote bone resorption, whereas Tregs suppress inflammation and facilitate tissue repair [158,159,160,161]. CDs may indirectly influence the differentiation and function of these T cell subsets by modulating the immune microenvironment. However, relevant studies of periodontitis remain limited. Tomić et al. [153] used multiple in vitro cell models to demonstrate that graphene quantum dots (GQDs) targeted monocyte-derived dendritic cells (DCs), modulated their differentiation and function, and indirectly inhibited pro-inflammatory T cell responses, including Th17 polarization.

## 4. Conclusions and Future Perspectives

Periodontitis is characterized as a chronic inflammatory condition involving multiple interconnected pathological mechanisms, involving microbial dysbiosis, dysregulated immune responses, oxidative stress, and abnormal bone metabolism. The advent of CDs has opened new avenues for periodontal therapy, attributable to their nanoscale tunability, multi-functionality, and exceptional biocompatibility as a rising class of nanomaterials. This review systematically summarizes the therapeutic mechanisms of CDs for periodontitis, including antimicrobial and anti-biofilm activity, regulation of oxidative stress, modulation of macrophage polarization, regulation of stem cell function, and regulation of bone homeostasis. These findings highlight the unique advantages of CDs in synergistically improving the periodontal immune microenvironment through multi-target and multi-pathway mechanisms.

While CDs demonstrate significant therapeutic potential for periodontitis, multiple barriers persist in their transition to clinical application: first, the unclear long-term biosafety and metabolic behavior of CDs in vivo. Factors such as synthesis methods, surface modifications, and particle size may affect their clearance and potential immunogenicity [162]. These issues must be further evaluated in large-animal studies with long-term follow-up. Second, the mechanisms by which CDs regulate the periodontal immune microenvironment are highly complex. Their biological functions are strongly influenced by intrinsic structural characteristics. However, the critical structure–function relationships remain poorly understood. To address this gap, future research should employ multi-omics strategies—including transcriptomics, proteomics, and metabolomics—to decipher the complex regulatory landscape underlying periodontitis pathogenesis. These analyses should be conducted at the levels of gene expression, protein–protein interactions, and metabolic phenotypes. Collectively, these data will establish a robust conceptual framework for guiding the rational design of CD-based biomaterials.

In summary, CDs as multifunctional nanoplatforms still require ongoing refinement and optimization for effective application in periodontitis therapy. With continued advances in material design and deeper mechanistic understanding, CDs are anticipated to overcome the limitations of conventional treatments. They hold strong potential to enable integrated therapeutic strategies that combine antimicrobial effects, immune modulation, and tissue regeneration, thereby offering a promising clinical approach for periodontal tissue repair and oral health maintenance.

## Figures and Tables

**Table 1:** Carbon dots (CDs) for antimicrobial and microbiome modulation.

Refs.	CDs	Precursors	Synthesis Method	Mechanism	Effect
[82]	BiCD-Ber	Berberine, potassium bismuth citrate, citric acid	Hydrothermal method	Bacterial metabolism	Bismuth ions neutralize *P. gingivalis* gingipain virulence factor, inhibiting pathogenicity.
[83]	Cu-CDs	Guanidine HCl, citric acid, copper chloride	Hydrothermal method	Positive charge & ROS generation	Positive charge enhances affinity for lipopolysaccharide (LPS)/peptidoglycan (PGN); oxidase (OXD)/peroxidase (POD)-like activity promotes reactive oxygen species (ROS) generation for antibacterial effects.
[84]	CQDSpds	Spermidine (Spd), CQDs	Pyrolysis method	Positive charge	Strong interaction with bacterial membranes due to super-cationic surface.
[85]	MCDs	Melatonin	Hydrothermal method	ROS generation & bacterial metabolism	Promotes bacterial ROS generation, disrupts membranes and biofilms; inhibits secA2 protein expression, affecting virulence factor secretion and survival.
[86]	GQD-Cur	Graphene oxide (GO), KMnO_4_, Curcumin	Microwave method	PDT	Blue LED light excitation reduces *Porphyromonas gingivalis (P. gingivalis)*, *Aggregatibacter actinomycetemcomitans (A. actinomycetemcomitans)* and *Prevotella intermedia (P. intermedia)* colonies and pathogen load in mixed biofilms.
[87]	Cu-RCDs-C35	Cu-RCDs, cocoamidopropyl betaine (CAB-35)	-	PTT	Good photothermal performance under 808 nm laser (29.51% efficiency), 99.36% and 99.98% antibacterial rate against *Escherichia coli* (*E. coli)* and *Staphylococcus aureus* (*S. aureus)*.
[88]	L-C-dots	*Lactobacillus acidophilus* *(* *L. acidophilus* *)*	Hydrothermal method	Synergistic antibacterial	Combines with antibiotics, reduces minimum inhibitory concentration (MIC), enhances drug stability, solubility, biocompatibility, permeability.
[89]	CD-AMX	Wheat bran	Hydrothermal method	Synergistic antibacterial & Delivery	Amoxicillin (AMX) delivery carrier, high loading & sustained release; antibacterial against Gram-positive and Gram-negative bacteria.

**Table 2:** CDs for antioxidant.

Refs.	CDs	Precursors	Synthesis Method	Mechanism	Effect
[15]	CDs	Citric acid, ethane diamine	Hydrothermal method	Enzyme-like activity	Exhibits dismutase SOD-like and catalase CAT-like activities, scavenging O_2_•^−^ and H_2_O_2_, reducing ROS in human umbilical vein endothelial cells (HUVECs).
[59]	MT-CDs	Melatonin	Hydrothermal method	Direct free radical scavenging	Inherits potent antioxidant properties of melatonin; modulates intracellular ROS levels.
[83]	Cu-CDs	Guanidine hydrochloride, citric acid, copper chloride	Hydrothermal method	ROS inhibition	Decomposes low concentrations of H_2_O_2_ into H_2_O and O_2_.
[85]	MCDs	Melatonin	Hydrothermal method	Signaling pathways	Upregulates expression of antioxidant genes *Sirt1* and *Nrf2*.
[101]	CD-PB-TPP	Polyethyleneimine-600, citric acid, K_4_[Fe(CN)_6_]·3H_2_O, Triphenylphosphine	Hydrothermal method	Enzyme-like activity	SOD and CAT-like activities scavenge ROS, reverse mitochondrial dysfunction, inhibit senescence.
[105]	DCDs	Citric acid, ammonium fluoride, dexamethasone	Hydrothermal method	Enzyme-like activity	Possesses SOD-like activity scavenging ROS (•OH) while retaining dexamethasone’s activity.
[106]	NAC-CPDs	Citric acid, N-acetyl-L-cysteine	Solvothermal method	Signaling pathways	Scavenges ROS, modulates Keap1/Nrf2 pathway to regulate redox homeostasis in periodontitis.

**Table 3:** CDs for regulating stem cell functions.

Refs.	CDs	Precursors	Synthesis Method	Mechanism	Effect
[15]	CDs	Citric acid, ethane diamine	Hydrothermal method	Vascularization	Enhances HUVEC tube formation in vitro, up-regulates VEGFA secretion, alleviates endothelial dysfunction.
[59]	MT-CDs	Melatonin	Hydrothermal method	Anti-inflammatory & Signaling pathways	Scavenges ROS, maintains mitochondrial homeostasis, inhibits inflammatory mediator production, activates Nrf2/HO-1 pathway, reduces *Bax* and *Caspase-3* expression and inhibits apoptosis.
[34]	CEWH	Citric acid, urea, raw egg white	Solvothermal method	Macrophage polarization	Promotes hair follicle regeneration via M2 macrophage polarization, creating a pro-repair microenvironment.
[85]	MCDs	Melatonin	Hydrothermal method	Proliferation & Differentiation	Promotes bone marrow stromal cells (BMSCs) osteogenic differentiation, upregulates osteogenesis-related genes (*Alpl*, *Runx2*, *Col1a1*) and proteins (RUNX2, OPN, COL1).
[105]	DCDs	Citric acid, ammonium fluoride, dexamethasone	Hydrothermal method	Proliferation and differentiation	Induces osteogenic differentiation of BMSCs under inflammation and enhances mineralization.
[105]	DCDs	Citric acid, ammonium fluoride, dexamethasone	Hydrothermal method	Macrophage polarization	Promotes M1 to M2 transition via ROS clearance.
[124]	CPP-CDs	Purple sweet potato tubers	Hydrothermal method	Anti-inflammatory	Inhibits cytokine secretion, reduces ROS, suppresses TLR4/NF-κB pathway and NLRP3 inflammasome activation in macrophages.
[124]	CPP-CDs	Purple sweet potato tubers	Hydrothermal method	Macrophage polarization	Inhibits pro-inflammatory cytokines (*IL-1β*, *IL-6*, *TNF-α*), inhibits pyroptosis, promotes macrophage polarization from M1 to M2 phenotype.
[125]	PCC-CDs	Phellodendri chinensis cortex	Calcination method	Macrophage polarization	Inhibits *TNF-α*, *IL-6*, *IL-17A*, *IL-23* expression; promotes M2 polarization while inhibiting M1 polarization.
[126]	CDs	Folic acid	Hydrothermal method	Macrophage polarization	Inhibits activation of NF-κB and MAPK signaling pathways, reprograms macrophage polarization.
[127]	MCDs	Metformin HCl, carboxymethyl chitosan	Hydrothermal method	Proliferation and differentiation	Alleviates inflammation via PI3K/AKT pathway, promotes osteogenic differentiation of human periodontal ligament stem cells (hPDLSCs) under lipopolysaccharide (LPS)-induced inflammation.
[128]	CDots	Polyethyleneimine, ascorbic acid	Hydrothermal method	Macrophage polarization	Binds PI3K catalytic subunit (PIK3CD), inhibiting PI3K/AKT/mTOR pathway and enhancing M1 polarization.
[129]	CDs	Citric acid	Hydrothermal method	Proliferation and differentiation	Promotes rBMSCs osteogenic differentiation via ROS-mediated MAPK pathway, enhances mineralization, allows tracking.
[130]	AACDs	Aspirin, adenosine	Hydrothermal method	Proliferation and differentiation	Promotes hBMSCs osteogenic differentiation; more effective than adenosine or aspirin alone.
[131]	C/N-GQDs-PEI -PLGA/pZNF	C-GQDs, N-GQDs, branched polyethyleneimine, carbodiimide hydrochloride, N-hydroxysuccinimide, carboxylated poly (lactic-co-glycolic acid)	-	Vascularization	Provides good HUVECs adhesion and growth conditions; delivers *pZNF580* gene, promoting HUVECs proliferation and migration.
[78]	Cur-Alen CDs	Alendronate, curcumin	Hydrothermal method	Proliferation and differentiation	Anti-inflammatory and antioxidant effects, promotes *Runx2*, *Alp* and *Opn* expression in LPS-stimulated BMSCs.
[132]	GQDs	-	-	Proliferation and differentiation	Promotes osteogenic differentiation of PDLSCs under LPS-induced inflammatory conditions.

**Table 4:** CDs for regulating bone homeostasis.

Refs.	CDs	Precursors	Synthesis Method	Mechanism	Effect
[126]	CDs	Folic acid	Hydrothermal method	Regulation of osteoimmunity	Inhibits NF-κB/MAPK pathways, modulates macrophage polarization and protects chondrocyte function.
[78]	Cur-Alen CDs	Alendronate, curcumin	Hydrothermal method	Regulation of osteoimmunity & osteoclast inhibition	Reduces inflammatory cytokine levels in RAW264.7 cells, exerts pro-osteogenic activity in vivo and inhibits key osteoclast differentiation genes (*Nfatc1*, *Calcr*, *Ctsk*).
[153]	GQDs	Spectroscopic graphite rods	Electrochemical method	Regulation of osteoimmunity	Inhibits differentiation and maturation of human monocyte-derived DCs, indirectly suppresses pro-inflammatory T cell responses (e.g., Th17).
[154]	PCDs	Sour apple	Hydrothermal method	Osteoclast inhibition	Down-regulates osteoclast-related genes (*Chem*, *Chem23*, *NFATc1*, *ACP5*, *Ctsk*, *Itgb3*); up-regulates *SIRT1*, reducing mouse osteoclast progenitor proliferation.
[11]	MCDs	Metformin HCl, citric acid	Hydrothermal method	Osteoblast promotion	Induces rBMSCs osteogenic differentiation via ERK/AMPK pathway, more effective than metformin.
[155]	Nar-CuCDs	Ammonium citrate, naringin, copper chloride	Hydrothermal method	Osteoblast promotion & regulation of osteoimmunity	Enhances osteogenic capability and mineralization of rBMSCs, optimizes osteoimmune microenvironment via ROS scavenging and M2 polarization, promoting rBMSC osteogenic differentiation.
[132]	GQDs	-	-	Osteoblast promotion	Activates Wnt/β-catenin signaling, upregulates osteogenesis-related genes (*ALP*, *RUNX2*, *OCN*), increases mineralized nodule formation.

## Data Availability

No new data were created or analyzed in this study. Data sharing is not applicable to this article.

## Abbreviations

The following abbreviations are used in this manuscript:

*A. actinomycetemcomitans*	*Aggregatibacter actinomycetemcomitans*
CAT	catalase
CDs	carbon dots
CNDs	carbon nanodots
–COOH	carboxyl
CPDs	carbonized polymer dots
CQDs	carbon quantum dots
DFSCs	dental follicle stem cells
GOQDs	graphene oxide quantum dots
GPx	glutathione peroxidase
GQDs	graphene quantum dots
HUVECs	human umbilical vein endothelial cells
LPS	lipopolysaccharide
MIC	minimum inhibitory concentration
MSCs	mesenchymal stem cells
–NH_2_	amino
NIR	near-infrared
–OH	hydroxyl
OMVs	outer membrane vesicles
OXD	oxidase
*P. gingivalis*	*Porphyromonas gingivalis*
*P. intermedia*	*Prevotella intermedia*
PDLSCs	periodontal ligament stem cells
PDT	photodynamic therapy
PGN	peptidoglycan
PL	photoluminescence
POD	peroxidase
PS	photosensitizer
PTT	photothermal therapy
rBMSCs	rat bone marrow mesenchymal stem cells
ROS	reactive oxygen species
SOD	superoxide dismutase
UV–Vis	ultraviolet–visible
